# Factors Determining the Antimicrobial Effectiveness of Chitosan: A Critical Analysis of the Impact of Molecular Weight and Degree of Deacetylation

**DOI:** 10.3390/molecules31142412

**Published:** 2026-07-09

**Authors:** Karolina Czajkowska, Maciej Rybicki, Karol Kamil Kłosiński, Radosław Aleksander Wach

**Affiliations:** 1 Department of Biomedicine and Experimental Surgery, Faculty of Medicine, Medical University of Lodz, Narutowicza 60, 90-136 Lodz, Poland; karolina.czajkowska@umed.lodz.pl; 2 Plastic, Reconstructive and Aesthetic Surgery Clinic, Institute of Surgery, Medical University of Lodz, Dr. Stefana Kopcinskiego 22, 90-153 Lodz, Poland; maciej.rybicki@stud.umed.lodz.pl; 3 Institute of Applied Radiation Chemistry, Faculty of Chemistry, Lodz University of Technology, Wroblewskiego 15, 93-590 Lodz, Poland

**Keywords:** chitosan, molecular weight (MW), degree of deacetylation (DD), antimicrobial activity, antibacterial, antifungal, in vitro, MIC

## Abstract

Despite chitosan’s proven biocidal potential, there is a lack of consensus regarding the influence of its key physicochemical parameters, degree of deacetylation (DD) and molecular weight (MW), on its antimicrobial efficacy. The aim of this study was to quantitatively synthesize available literature data and provide an exploratory statistical analysis of the trends regarding the influence of chitosan DD and MW on the minimum inhibitory concentration (MIC). A PRISMA-guided literature review (2016–2026) across four major databases extracted 127 independent in vitro experiments, Gram-positive/negative bacteria and fungi. Physicochemical correlations were analyzed using multiple linear regression. Insoluble polymer fractions (here set as DD < 60%) critically interfere with MIC determination due to restricted diffusion and were strictly excluded from analyses. For the optimized group of soluble chitosans (*n* = 106, R^2^ = 0.36), DD was the dominant factor determining biocidal activity (*p* < 0.0001)—every 1% increase in DD reduces the logMIC by an average of 0.040. In contrast, molecular weight did not emerge as a statistically significant predictor within this model (*p* = 0.156), suggesting that the degree of deacetylation remains the primary driver of antimicrobial efficacy among soluble chitosans. In summary, maximizing chitosan antimicrobial activity requires high DD polymers, and rigorous standardization (excluding insoluble fractions) is essential for the proper design of new biomaterials.

## 1. Introduction

### 1.1. Chitosan: An Important Natural Polysaccharide

#### 1.1.1. From Chitin to Chitosan—Origin and Structural Composition

Chitin is the second most abundant natural polymer after cellulose, occurring naturally as structural material in the shells of marine crustaceans, the exoskeleton of arthropods, the cell walls of fungi, and even in some invertebrates [1,2,3,4]. The first references confirming its isolation by boiling chitin in a concentrated solution of potassium hydroxide (KOH) date back to 1859 [5,6]. However, while Rouget was the first to isolate this substance, he did not coin its moder name. The term “chitosan” was introduce decades later, in 1894, by the scientist Felix Hoppe-Seyler [7]. Chitin is a linear polysaccharide composed of glucose-derived monomers: 2-N-acetyl-D-glucosamine, linked by β-1,4 bonds. It is worth noting that chitin can naturally occur in three allomorphic forms: most commonly as alpha (α) and beta (β), and less commonly as gamma (γ), which is a structural hybrid of α and β forms [5,8].

**Figure 1:**
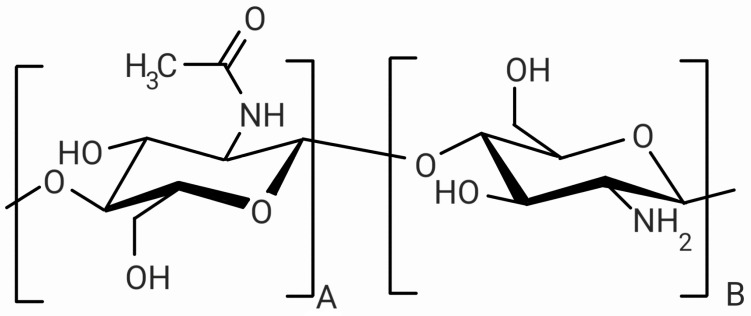
Chemical structure of chitosan. The repeating monosaccharide units are represented by N-acetyl-2-amino-2-deoxy-D-glucose (**A**) and 2-amino-2-deoxy-D-glucose (**B**). Chitosan: A < B.

The enzyme chitin deacetylase (CDA) catalyzes the process of removing acetyl groups (deacetylation) from N-acetylglucosamine residues, leading to the formation of chitosan with varying degrees of deacetylation (DD) [9]. Enzymatic deacetylation, in particular when carried out under the influence of fungal CDA, allows the production of chitosan with a significantly high degree of deacetylation and controlled molecular weight. Chitosan is obtained from chitin through alkaline deacetylation, typically by treating crustacean shell-derived chitin with a 40–50% sodium hydroxide (NaOH) or potassium hydroxide (KOH) solution at temperatures above 100 °C [10,11]. This process converts acetamide groups in chitin into amine groups, requiring subsequent washing and drying to produce chitosan. Then, as a result of the removal of acetyl groups from N-acetylglucosamine units, new glucosamine units are formed [5]. Deacetylated chitin is referred to as chitosan when the degree of deacetylation, i.e., the percentage of restored amino groups (−NH_2_), exceeds 50%. The chemical structure of chitin and chitosan is illustrated in Figure 1.

#### 1.1.2. Characteristics of Chitosan

Chitosan has attracted widespread interest in the scientific world due to its unique qualities—biocompatibility and antimicrobial properties, as well as antioxidant and hemostatic effects—and consequently, its use has been established in various fields of medicine [12,13,14]. Specifically, its antimicrobial properties have enabled chitosan to be used in modern wound dressings, which still remains an important area of research interest to this day. A correlation among molecular weight (MW) and degree of deacetylation (DD) of chitosan and its antimicrobial properties has been implied; nevertheless, the literature data on this issue are highly divergent [15]. For example, according to Zheng and Zhu, 2003, low MW corresponds to increased activity against Gram-negative bacteria (mainly *E. coli*), while high MW is, in their view, more beneficial for combating Gram-positive bacteria (*S. aureus*) [16,17].

It should be mentioned that a higher degree of deacetylation (DD) is associated with a greater number of positively charged amino groups (−NH_3_^+^) along the chitosan macromolecule. This allows for stronger interaction with the negatively charged surface of microorganism cells, which results in greater antimicrobial properties [16,18,19,20]. However, the optimal combination of MW and DD values for which antimicrobial activity would reach its maximum has not yet been defined.

### 1.2. The Antibiotic Crisis—Looking for Alternatives to Classic Antibiotic Treatment

According to the WHO, drug resistance is considered one of the key threats to public health [21]. Furthermore, the Food and Drug Administration (FDA) approved only 17 systemic antibiotics between 2010 and 2021. It should be emphasized that the vast majority of newly introduced pharmaceuticals are only modifications of existing drugs, rather than innovative active substances with a new mechanism of action [22]. Despite the growing problem of antimicrobial resistance (AMR) the number of new products approved is dramatically low. Furthermore, in 2021–2022, FDA and European Medicines Agency (EMA) did not approve a single completely new antibiotic. In the last 5 years, most newly introduced preparations are not new classes of drugs, but modifications of older structures (mainly cephalosporins) or combinations of old antibiotics with new β-lactamase inhibitors [23,24,25]. Growing multidrug resistance (MDR), hospital-acquired infections, and the emergence of “superbugs” have indirectly contributed to increased morbidity and mortality. It has also been shown that infections caused by MDR bacteria often result in emergency and early treatments failure, which leads to longer hospitalization and thus to a significant increase in the healthcare cost. The World Bank estimates that increasing antimicrobial resistance could result in additional healthcare costs of $1 trillion by 2050 [21,26].

### 1.3. Identification of Research Gaps and the Objective of the Study

Despite the large number of publications and growing interest in the issue, scientific literature is characterized by considerable heterogeneity in terms of study design, methodology, chitosan extraction and preparation methods, analytical techniques, and reporting of results. Furthermore, the literature contains many contradictory conclusions. Lack of standardization, including insufficient reporting of chitosan synthesis processes and research methods used, combined with contradictory results, makes it difficult to draw clear conclusions. The authors of publications often omit essential data on the properties of the biopolymer used, such as MW, DD, or details of the production and processing, even though the established methodologies are available, see for instance [27]. This makes it problematical to broadly compare the results of individual microbiological studies and reliably evaluate their credibility. Moreover, some studies omit important physicochemical parameters (such as temperature, incubation time, pH, and type of medium), and sometimes do not even provide satisfying information on the species of organism used to assess antimicrobial properties. Therefore, it is crucial to examine the antimicrobial properties of chitosan more thoroughly, determine the MW and DD ranges with optimal activity, and ultimately implement products based on this material into common clinical practice. This constitutes a serious limitation in the interpretation of results and hinders the conduct of reliable systematic reviews and meta-analyses. These observations impelled the authors of this paper to arrange a narrative literature review. This study systematically, critically and concisely analyzes the current scientific literature, presenting the results—the influence of chitosans’ degree of deacetylation and molecular weight on antimicrobial properties—in a comparative and visual manner, and also identifies trends and directions in which new, more methodologically refined research on the antimicrobial properties of chitosan should proceed.

## 2. Materials and Methods

### 2.1. Literature Review Draft

**Figure 2:**
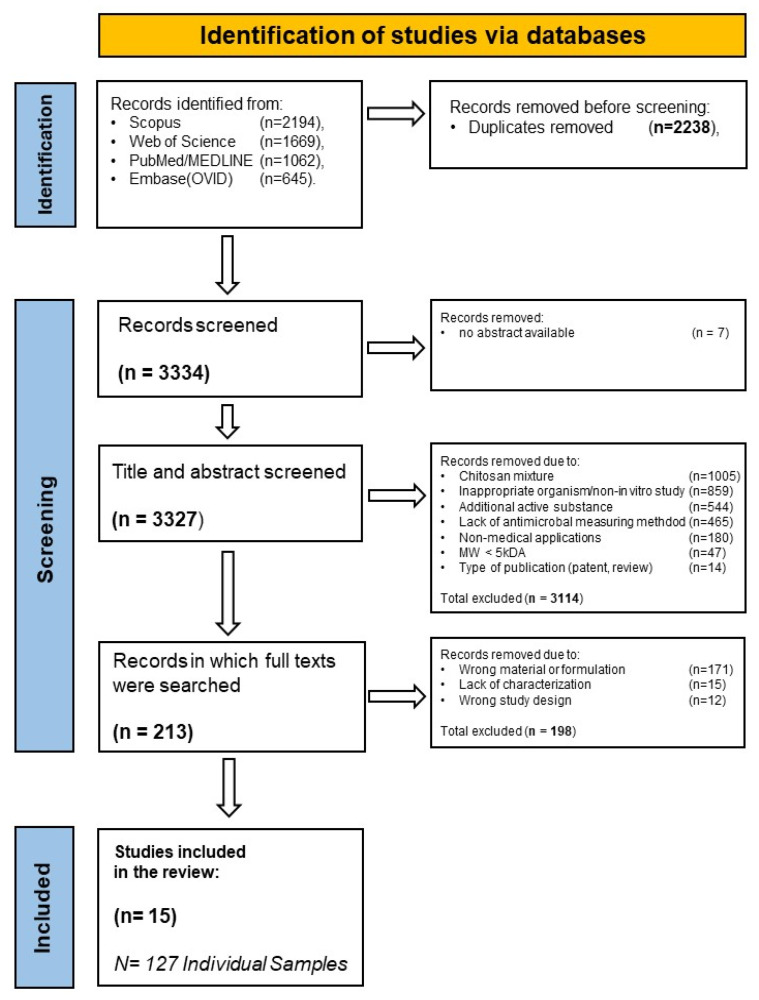
Flow diagram illustrating the identification and selection of articles.

A narrative literature review with elements of systematic searching was undertaken to identify and critically analyze studies with a particular concentration on the antimicrobial nature of chitosan relative to its molecular weight (MW) and degree of deacetylation (DD). The following review is exploratory and comparative in nature, and its aim is to establish a research group consisting of the most methodologically homogeneous in vitro studies, enabling correlations to be drawn between the physicochemical parameters of chitosan, namely DD and MW, and the observed antimicrobial activity. Due to the high heterogeneity of the analyzed data and the nature of the paper, a formal systematic review (with PROSPERO registration) was not conducted; however, a strictly structured procedure for identifying, selecting, and extracting data was applied. The process of identifying and selecting publications is presented in the form of a PRISMA-style flow diagram—Figure 2.

Identification and selection of publications for this narrative review with meta-analysis was conducted in accordance with the PRISMA scheme, covering four main databases: Scopus, Web of Science, PubMed/MEDLINE, and Embase. After removing 2238 duplicates, 3334 records were initially scanned. Then, in a two-stage selection process involving analysis of titles and abstracts, followed by subsequent evaluation of 213 full texts, studies that did not meet the criteria were rejected for reasons such as a lack of an appropriate MIC determination methodology, the use of chitosan mixtures or antimicrobial additives, or incomplete and flawed physicochemical characterization of the polymer. Finally, 15 articles were selected for narrative synthesis, from which a total of 127 individual research trials (*n* = 127) were extracted and subsequently subjected to assessment. Among them, 106 samples that met all quality and methodological criteria were included in the quantitative synthesis, which enabled detailed statistical modelling to be carried out.

### 2.2. Timeframe and Databases Analyzed

The systematic literature search covered studies published in specialist literature over the last 10 years, analyzing scientific articles from 1 January 2016 to 1 February 2026. The search was conducted in the following databases: PubMed/MEDLINE, Scopus, Embase (OVID), and Web of Science Core Collection.

**Figure 3:**
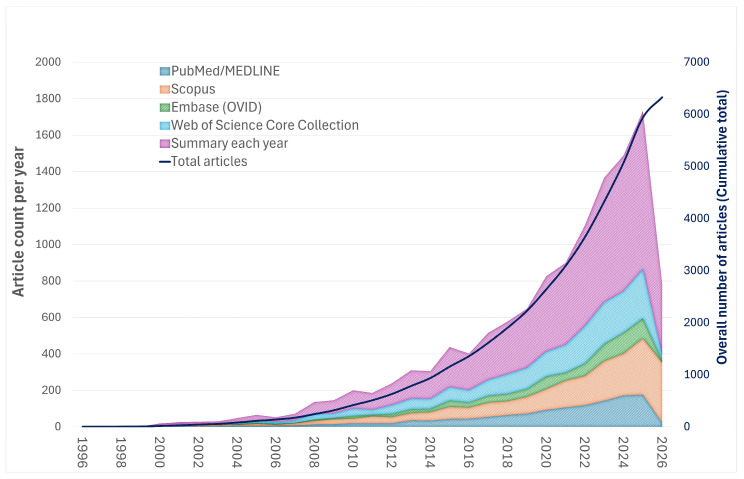
Trends in publications on antimicrobial chitosan research (1996–2026). The colored stacked areas illustrate the annual article count extracted from individual databases (PubMed/MEDLINE, Scopus, Embase, and Web of Science Core Collection), with the top purple envelope (Summary each year) representing the total annual publication output, which is plotted against the left Y-axis. The solid black line (Total articles) illustrates the cumulative growth of the combined dataset over the 30-year period, plotted against the right Y-axis (Cumulative total).

The chronological scope was chosen in order to take into account contemporary research on chitosan, which was carried out using relatively modern manufacturing and analytical methods. The authors also noticed a significant increase in interest in chitosan and its antimicrobial properties over the last decades. To capture the trend, the dynamics of publications between 1996 and 2026 are presented in Figure 3, a composite chart. The left axis shows the annual number of articles in individual databases, while the line (right auxiliary axis) shows the total cumulative number of publications in the period under study. The analysis shows a marked exponential increase in interest in chitosan in scientific literature.

### 2.3. Search Strategy

The search strategies were based on a combination of keywords, synonyms, and controlled terms (e.g., MeSH), including (i) chitosan and its synonyms, (ii) antimicrobial activity, (iii) bacteria and/or fungi, and (iv) physicochemical parameters (DD, MW).

Search strategies were tailored for each database, taking into account the syntax and indexing system. Logical operators were used to narrow down the results to in vitro studies on “pure chitosan” and to exclude publications on chitosan with additives in the form of nanoparticles or ions (e.g., silver, gold), antibiotics or fungicides, composites, and other substances whose addition may have a significant effect on antibacterial or antifungal activity. The complete search strategy is presented in Supplementary Materials Table S1.

Furthermore, a manual search of the reference lists of publications eligible for review (backward snowballing) was conducted to identify additional articles that met the inclusion criteria for the study.

The following search restrictions were applied: (i) articles published only in English, (ii) full texts available, and (iii) publication in peer-reviewed scientific journals.

### 2.4. Eligibility Criteria

Only studies in the form of original articles presenting authors’ own research data, meeting the following eligibility criteria, were included in this narrative review with elements of a systematic literature review: (i) in vitro experimental study, (ii) evaluation of antimicrobial activity against bacteria (Gram-positive or Gram-negative) or fungi, (iii) use of “pure chitosan,” which we define as a polysaccharide that is not chemically modified and not enriched with additional substances with potential antimicrobial activity, (iv) clearly defined molecular weight (MW) in the form of a specific numerical value or convertible range, (v) clearly defined degree of deacetylation (DD) or degree of acetylation (DA), (vi) chitosan molecular weight ≥ 5 kDa, (vii) the minimum inhibitory concentration (MIC) test was used, and (viii) available in English.

Meanwhile, publications were excluded if: (i) chitosan with a molecular weight of less than 5 kDa (e.g., nanochitosan) was used, (ii) chitosan was combined with a substance with potential antimicrobial properties (antibiotic, Ag ions, natural substances with antimicrobial significance), (iii) MW, DD/DA, or both variables were missing, (iv) reliable antimicrobial activity tests were not performed, (v) the studies were in vivo, (vi) viruses, protozoa, or other organisms that were not bacteria or fungi were analyzed, (vii) the publication was available in a language other than English, or (viii) the publication was a review article, conference proceeding, or grey literature.

### 2.5. Article Selection Process

All records obtained in the database search process were exported to the bibliography management program Mendeley Cite, where deduplication was performed. This was followed by a two-stage selection process:

1. Analysis of titles and abstracts.
2. Analysis of full texts.

The selection was carried out by two independent reviewers (K.C. and M.R.). In case of any discrepancies, decisions were made by a third reviewer (K.K.K.). The entire selection process is presented in the PRISMA flow diagram (Figure 2).

### 2.6. Data Extraction

Data from publications included in this narrative literature review were extracted into a structured spreadsheet by two independent reviewers. Any discrepancies were reviewed and reconciled until consensus was reached.

The following variables were analyzed: (i) molecular weight of chitosan (MW), (ii) degree of deacetylation (DD) or acetylation (DA), (iii) form and purity of chitosan, (iv) presence of additives, (v) microorganism species, (vi) selected method of antimicrobial activity, (vii) whether a test for evaluating antimicrobial properties other than the MIC test was used, (viii) experimental conditions (including pH, temperature, incubation time, and medium), (ix) chitosan concentration, and (x) methods for determining MW and DD (DA).

### 2.7. Data Normalization and Transformation

If DD was specified in the study, the original value was entered as a decimal fraction. It is worth noting that in some papers the authors used the degree of acetylation (DA) instead of the degree of deacetylation (DD); therefore, the following conversion was applied: DD = 1 − DA. DA specified as a percentage was converted to a decimal fraction before calculation.

All MW values were converted to kilodaltons (kDa, which corresponds to kg/mol in the SI system, where 1 Da = 1 g/mol). In the case of ranges, the minimum, maximum, and median values were used. Values given as Mn, Mw, Mv, or other types of averages were retained in their original form and interpreted with caution.

Data consistency was checked after conversion. Inconsistent, ambiguous, or unobtainable values were marked in the spreadsheet and included only in descriptive form.

### 2.8. Grouping and Analysis

The research group was divided according to regna and Gram staining into: (a) Gram-positive bacteria, (b) Gram-negative bacteria, and (c) fungi. The analysis was exploratory in nature and included an assessment of the relationship between MW, DD, and MW × DD and antimicrobial activity. Data visualization (bubble charts, heat maps—see below) was used. Due to the heterogeneity of the methods and the data obtained, no formal meta-analysis was performed.

### 2.9. Assessment of Research Quality (Risk of Bias)

A formal assessment of publication bias was not performed. Instead, methodological quality was assessed narratively using a proprietary checklist covering key elements of the research experiment (including MW, DD, pH, temperature, chitosan concentration, experimental method, and control conditions).

The classification was based on the percentage of control elements met. The studies were divided into: (i) high (≥70%), (ii) medium (50–69%), and (iii) low quality (<50%). The quality assessment was not a formal criterion for exclusion from the review, but it did influence the interpretation of the results.

### 2.10. Quantitative Synthesis and Statistical Modelling

Quantitative synthesis and statistical modelling were performed using Microsoft Office LTSC Professional Plus 2024 (with the Analysis ToolPak module) and Statistica 8 software. Statistical tests used were as follows.

- Pearson’s linear correlation analysis (heat map).
- Multiple linear regression:
  - Snedecor’s F-test (Analysis of Variance—ANOVA), F = 1.71 × 10^−11^ < 0.001, highly significant for *n* = 108.
  - Student’s *t*-test (for the regression coefficient).
    MWmean *p*-value = 0.0293 < 0.05; statistically significant.
    DD% *p*-value = 5.63 × 10^−12^ < 0.05; highly significant.
    Intercept *p*-value = 2.91 × 10^−27^ < 0.05; highly statistically significant.

Given the considerable methodological heterogeneity of the included studies, formal meta-analysis was replaced by rigorous data exploration and predictive modelling. The level of statistical significance was set at *p* = 0.05.

**Figure 4:**
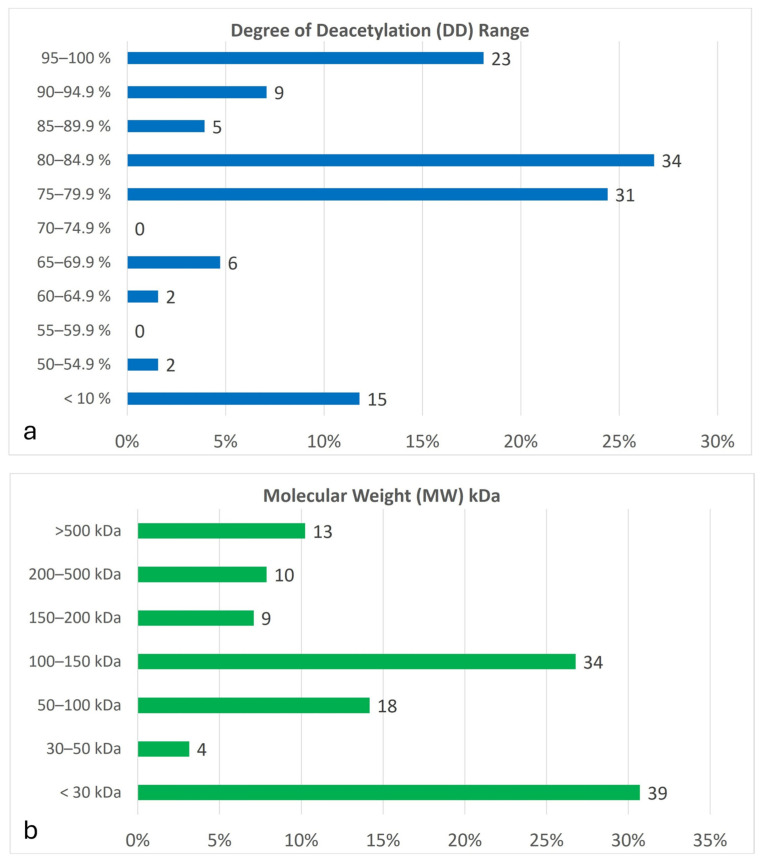
Distribution of specific ranges of deacetylation degree (**a**) (in percentage, upper diagram) and molecular weight (**b**) (in kg/mol = kDa, lower diagram) in the analyzed sample population. Bar length reflects the percentage share of a particular group in the total analyzed pool (*n* = 127), and the numerical values at the ends of the bars represent the number of samples (*n*) within a given range.

**Figure 5:**
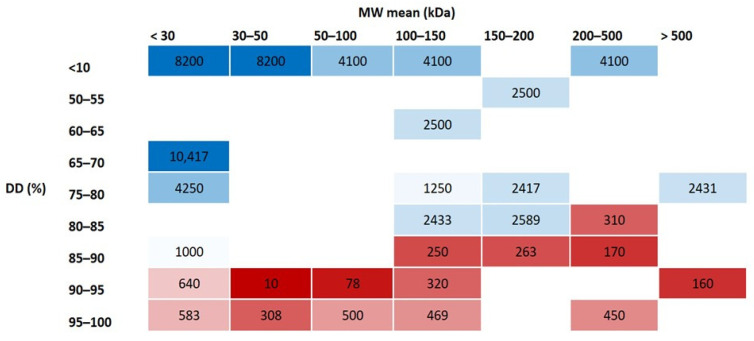
Global trends in the antimicrobial activity of chitosan depending on its physicochemical properties (MW & DD)—heatmap visualization (*n* = 127). Heatmap representing mean MIC values in mg/L. The lower MIC values (highest antimicrobial activity) are marked in red, while the highest MIC values (lowest antimicrobial activity) are highlighted in blue. The remaining white areas indicate a lack of data—no samples were found in the analyzed population for a given DD and MW. MIC values were entered in the appropriate fields. For clarity, the articles were grouped according to DD (%) and MW (kDa).

**Figure 6:**
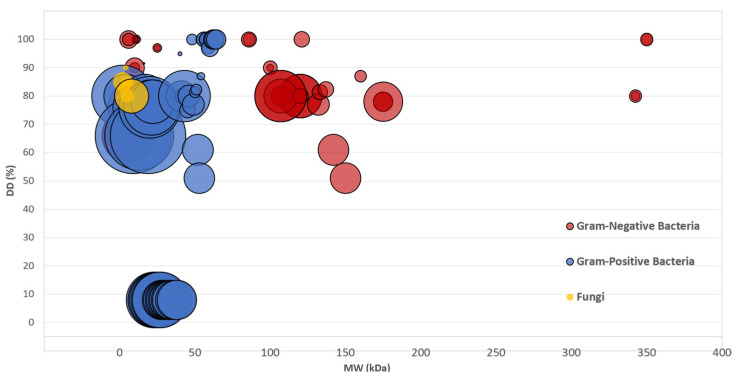
Three-dimensional distribution of chitosan activity: Effect of MW and DD on MIC, accounting for group specificity (Gram-positive bacteria vs. Gram-negative bacteria vs. fungi). Bubble chart illustrating the correlation between the degree of deacetylation (DD in %) and molecular weight (MW in kDa) with respect to MIC value, with smaller bubbles representing greater antimicrobial activity (lower MIC). Data points divided into three groups according to organism type: blue—Gram-positive bacteria, red—Gram-negative bacteria, and yellow—fungi.

**Figure 7:**
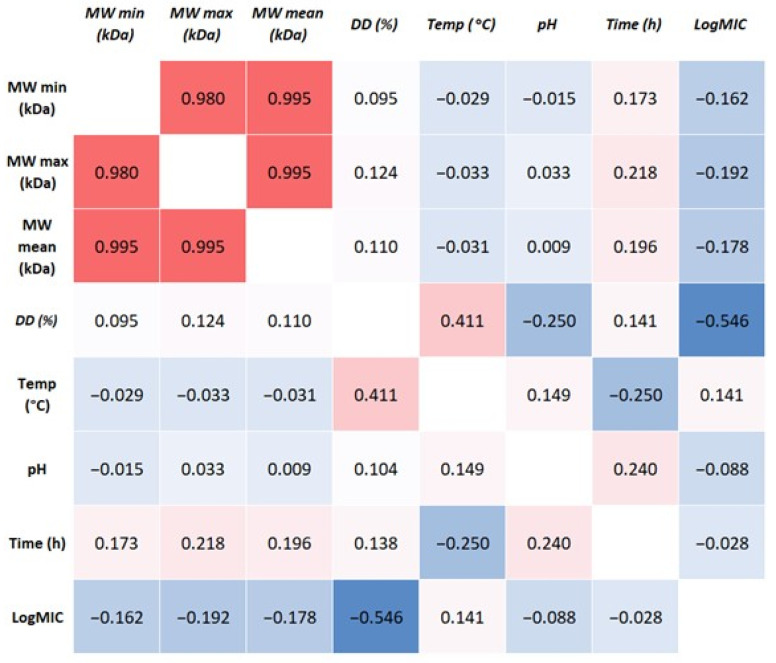
Visualization of correlations across variables (heatmap).

Based on the basic characteristics of the material shown in Figure 4, the subsequent analysis focuses on specific physicochemical parameters that affect the soluble fraction. During the first stage, the overall relationships between variables were assessed using Pearson’s correlation matrix and visualization in the form of heat maps (Figures 5 and 7) and three-dimensional bubble charts (Figure 6). This analysis allowed for a preliminary determination of the relationship between the physicochemical properties of chitosan and its antimicrobial activity.

**Figure 8:**
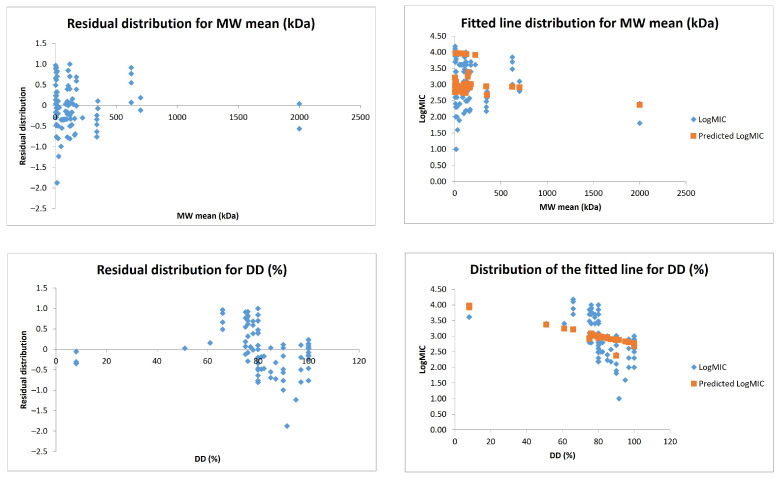
Series of diagnostic graphs of multiple regression for MW and DD (*n* = 127) showing residual distributions and the fitted line plots for both mean molecular weight (MW mean) and degree of deacetylation (DD). In the fitted line plots, the ‘Predicted LogMIC’ values represent the expected antimicrobial activity estimated by the multiple regression model for each sample, based on its specific combined parameters.

**Figure 9:**
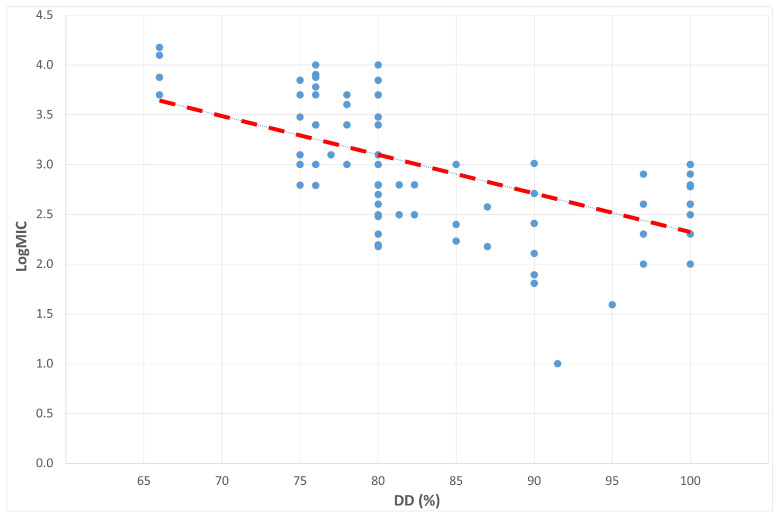
Scatter plot of DD (%) over logMIC with trend line (*n* = 106; Gram-negative = 53, Gram-positive = 46, Fungi = 7). Scatter plot showing the correlation between the logarithm of the minimum inhibitory concentration (logMIC) and the DD (%) value for the test sample (*n* = 106). The red dashed line represents a linear trend function, indicating a clear negative correlation between the analyzed parameters (an increase in the DD% value is associated with a decrease in the logMIC value).

**Figure 10:**
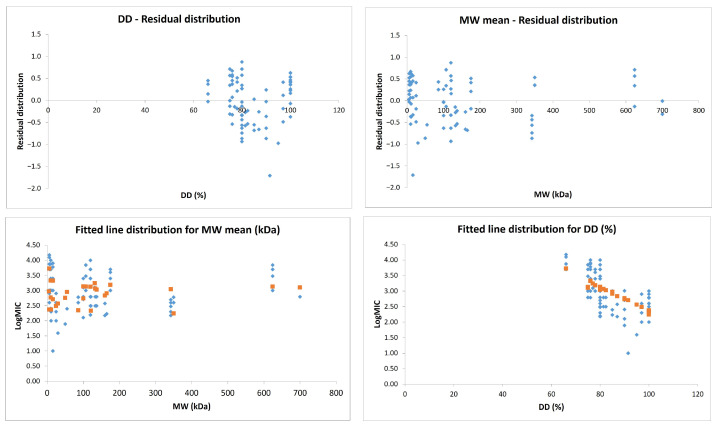
Series of diagnostic graphs of multiple regression for MW and DD (*n* = 106) showing residual distributions and plots with a fitted line for both MW mean and DD. In the plots with a fitted line, the “predicted logMIC” values reflect the predicted antibacterial activity estimated using a multiple regression model for each sample based on its specific, combined parameters.

The main research tool was multiple regression. To ensure the physicochemical correctness of the model, samples with chitin characteristics (DD < 60%) were excluded, and the final fit was performed for the soluble chitosan fraction (*n* = 106). The dependent variable was the logarithmic MIC value (logMIC), while the explanatory variables were the degree of deacetylation (DD) and the mean molecular weight (MW mean). The quality of the fit and the fulfilment of the model assumptions were confirmed on the basis of diagnostic graphs (Figures 8 and 10). The influence of DD on biocidal activity was illustrated by a scatter plot with a trend line (Figure 9).

## 3. Results

### 3.1. Structure of the Studied Microorganism Population

This narrative literature review included 15 individual original scientific articles that met all inclusion criteria, resulting in 127 individual records on chitosan with a specific molecular weight (MW) and degree of deacetylation (DD), tested in vitro on a selected organism, with the obtained MIC result of antimicrobial activity. Please refer to the Section 2 below.

Within the analyzed group of 127 cases, 120 involved bacteria, including 63 cases (49.6%) related to Gram-positive bacteria and 57 (44.9%) related to Gram-negative bacteria. Furthermore, the study included 7 samples (5.5%) relating to fungi. The classification of microorganisms is presented in Supplementary Table S2.

Of the samples tested, 31 (24.4%) were studies on *S. aureus*, 21 (16.5%) concerned *E. coli*, 8 (6.3%) *P. aeruginosa*, 6 (4.7%) *S. mutans*, 5 (3.9%) *B. subtilis*, and 5 (3.9%) *C. albicans*. A comprehensive quantitative and percentage analysis of the tested strains is presented in Table 1.

### 3.2. Impact of MW and DD on Antimicrobial Activity

Physical and chemical parameters analysis, with particular emphasis on MW and DD, revealed considerable diversity in chitosan samples used in the studies considered. Regarding the degree of deacetylation (DD), the most numerous group were those with high DD, in the ranges 80–84.9% (*n* = 35) and 75–79.9% (*n* = 31), which accounted for more than half of all samples tested. It should be noted that extreme values with very high DD: 95–100% (*n* = 23) also represented a substantial group, whereas samples with a low DD < 10% (*n* = 15) accounted for a minority in the following analysis. In terms of MW, we observed a clear trend in researchers’ preference for low molecular weight chitosans—the dominant group consisted of chitosans with a molecular weight of below 30 kDa (*n* = 39). The second largest group was in the medium molecular weight range of 100–150 kDa (*n* = 34), whereas chitosans with a high molecular weight (>500 kDa) were analyzed much less frequently (*n* = 13). The detailed breakdown of subpopulations is shown in Figure 4.

### 3.3. Demonstration of the Impact of Physicochemical Properties on Antimicrobial Activity

Multi-dimensional analysis of the gathered data was performed to obtain the relationships between MW, DD and the antimicrobial activity of chitosan. During the first stage, overall trends in the variability of minimum inhibitory concentration (MIC) were assessed in relation to the physicochemical parameters of the polymer. This allowed the identification of areas with the highest biocidal activity. The generated heat map (Figure 5) shows both the density of data and the intensity of the combined influence of MW and DD parameters on the effectiveness of the tested preparations.

Furthermore, taking into account the considerable diversity of the microorganism populations studied (Section 2.1), the analysis was extended to include the specific characteristics of individual microorganism groups. Due to fundamental differences in cell wall composition, a three-dimensional model of activity distribution was developed (Figure 6). This visualization enables simultaneous assessment of the interactions between MW, DD, and MIC, with a clear discrimination between Gram-positive, Gram-negative, and fungal microorganisms. Such a representation of data makes it easier to observe whether the preferred parameters of chitosan differ depending on the target microorganism type.

### 3.4. Statistical Analysis of Factors Determining the Highest Antimicrobial Activity

Precise values of correlation coefficients for the tested variables: MW min, MW max, MW mean, DD, temperature, pH, incubation time and logMIC are presented in Figure 7. Owing to the small correlation coefficients for pH (−0.087), temperature (0.14), and incubation time (−0.028), these factors were not further analyzed, as the insufficient number of published data did not allow for drawing constructive conclusions. Only MW mean (kDa), DD (%) and logMIC (antimicrobial activity indicator) were qualified for regression analysis.

Multiple regression analysis (*n* = 127, R^2^ = 0.32) yielded only the impact of DD on MIC as statistically highly significant (*p* < 0.0005). A negative regression coefficient of –0.011 supports the findings of several previous studies that the higher the degree of deacetylation, the stronger the antimicrobial activity (lower MIC) [15]. Detailed results of the multiple regression are presented in Figure 8. Comparison of predicted values (orange squares) with the actual observed logMIC values (blue diamonds) visualizes the model’s goodness-of-fit and confirms the primary influence of DD over MW on the predicted outcomes. To validate the multiple linear regression model, a residual analysis was performed, as illustrated in Figure 8. The residual plots against both independent variables (DD and MW) indicate that the fundamental assumptions of linear regression were reasonably met. The residuals are randomly and relatively evenly distributed around the horizontal zero line, suggesting sufficient homoscedasticity and a linear relationship between the predictors and logMIC. No severe systematic deviations, such as clear funnel shapes or non-linear patterns, were observed. Furthermore, the analysis did not reveal any extreme outliers that could artificially bias the regression coefficients, confirming the adequacy of the descriptive model for the analyzed global dataset.

Prior to determining the final regression model and plotting the trend line, it was necessary to introduce a key physicochemical distinction in the analyzed data set. For this reason, all samples with DD < 60% were excluded from further statistical analysis. The final trend fit was performed only for the soluble chitosan fraction (DD > 60%). It should also be emphasized that there is a methodologically related issue with samples for which DD is reported in the literature as 100%. From a physicochemical point of view, achieving absolutely complete deacetylation is questionable, except for specific oligosaccharides or materials synthesized in a controlled manner. After excluding the insoluble fraction, multiple regression analysis was performed again for a narrowed group of 106 samples. The analysis shows that the DD is still essentially more important for the antimicrobial activity of chitosan than the size of the molecules (MW).

Regardless of its potentially low value, the quality of the model (R^2^ = 36%) can be considered satisfactorily high, especially in the case of a heterogeneous research sample comprising various microbiological studies conducted on diverse organisms, such as Gram-positive and Gram-negative bacteria, as well as fungi. The remaining 64% of the variance is accounted for by factors not included in the analysis. These are, among others, microbial strain characteristics (e.g., cell wall architecture), additional polymer properties (chitosan origin, synthesis method, and degree of deacetylation), and methodological variability inherent to biological tests (random error in MIC and DD measurements).

The derived regression equation is as follows:

logMIC ≈ 6.35 − 0.040 × DD − 0.00038 × MW(1)

Statistically, each 1% increase in the DD decreases logMIC by 0.019. MW may also marginally reduce logMIC, and the refined analysis indicates that this effect is statistically significant, rather than coincidental. A graphical representation of the effect of DD (%) on logMIC, together with a trend line, is presented in Figure 9.

### 3.5. Statistical Assessment of Activity Predictors and Dualistic Mechanisms of Action

Eliminating insoluble chitosan fractions from the analysis allowed for developing a highly statistically significant multiple regression model (F = 31.68; *p* < 0.0001). The adjusted R^2^ value indicates that MW and DD account for over 36% of the overall variance in antimicrobial activity.

Analysis of independent variables confirmed the significant contribution of DD (*p* = 1.9 × 10^−11^ < 0.0001). Each 1% increase in DD led to a mean reduction in logMIC of 0.040 (95% CI: 0.029–0.050). Interestingly, extraction of the dataset from insoluble chitin also revealed that while the overall model is statistically significant, the individual effect of MW did not reach the conventional significance threshold (*p* = 0.156). Therefore, variations in MW within the analyzed global dataset do not independently predict logMIC changes, leaving the degree of deacetylation as the dominant factor. Therefore, based on the derived dependency, for exemplary chitosan of 100 kg/mol molecular weight, one may expect the logMIC values 3.51, 3.11 and 2.71 chitosans of DD 70%, 80% and 90%, respectively. Detailed results of the multiple regression are presented in Figure 10.

**Figure 11:**
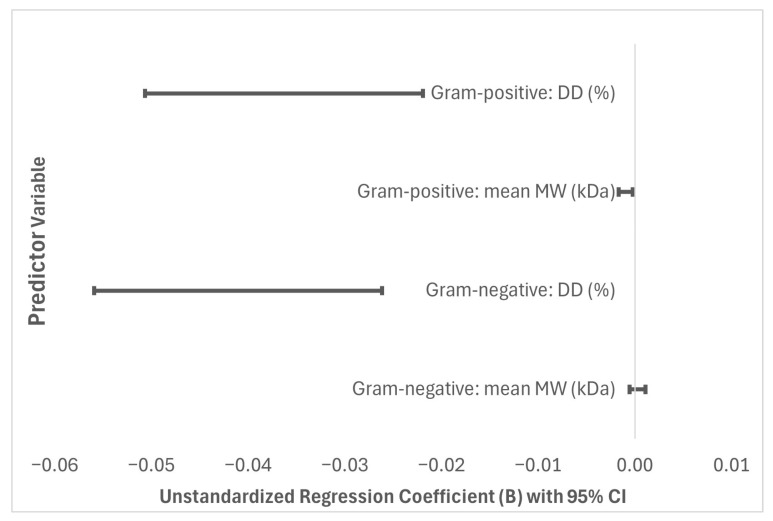
Forest plot of unstandardized regression coefficients (B) with 95% confidence intervals (CI) for the stratified bacterial models. A vertical reference line at the zero level indicates no effect (no statistical significance). Confidence intervals that cross the zero line indicate predictors of no significance (e.g., MW for Gram-negative bacteria), whereas those located entirely to the left of the zero line indicate a significant reduction in logMIC values (increased antibacterial efficacy).

Table 2 shows the predicted logMIC values, which resemble the experimentally determined values for the samples analyzed. As shown in Table 2, a linear trend is clearly observable for both independent variables. A higher degree of deacetylation (DD) consistently leads to a significant decrease in the predicted logMIC values across all analyzed molecular weights (MWs). Similarly, an increase in MW from 50 to 400 kDa further enhances the antimicrobial activity (lowers the logMIC), although the effect of DD appears to be more noticeable than that of MW within the tested ranges. The impact of chitosan parameters on antimicrobial activity, expressed as logMIC, along with the corresponding 95% confidence intervals, is illustrated in the forest plot in Figure 11.

Table 3 presents descriptive statistics for the bacterial dataset (*n* = 99, excluding fungi), highlighting specific differences between Gram-negative (*n* = 53) and Gram-positive (*n* = 46) strains. While the mean DD remains nearly identical for both groups (circa 83%), distinct variations are observed in MW and logMIC. Gram-positive bacteria exhibit a higher mean MW compared to Gram-negative strains (165.78 kDa vs. 147.4 kDa) and a broader range reaching up to 700.00 kDa (vs. 624.00 kDa). Furthermore, Gram-positive strains display slightly higher logMIC values, evident both in the mean (3.09 vs. 2.93) and the overall range (1.59–4.18 vs. 1.00–3.88).

## 4. Discussion

The increasing need for new antimicrobial agents is undeniable. Growing resistance to antimicrobial agents weakens or negates the effect of commonly used biocidal and biostatic substances, posing a serious threat to modern healthcare [28,29,30]. In this context, understanding the exact physicochemical parameters that dictate the efficacy of alternatives like chitosan is crucial.

Based on the definition, biopolymers with a degree of deacetylation below 60% exhibit properties characteristic of chitin rather than chitosan. Their primary characteristic is almost complete insolubility in standard aqueous solutions [3]. In the context of analyzing the results of in vitro microbiological tests, such as the determination of the minimum inhibitory concentration (MIC), insolubility means that there is practically no diffusion of the material into the medium. Consequently, the mechanism of interaction of this fraction with bacterial or fungal cells is physically incomparable to the action of soluble chitosan. Therefore, there is no justification for creating a common model for both groups, and doing so could introduce a specific methodological error.

Once the insoluble fraction has been removed, it becomes apparent that the diffusion is essential for biological systems. Such results strongly suggest that the free diffusion of macromolecules in the medium represents a critical baseline condition for observing a consistent statistical association regarding chitosan’s biocidal activity. Furthermore, analysis of the soluble fraction reveals a direct effect of the degree of deacetylation on logMIC. A higher DD, reflecting a greater number of free amino groups, strengthens the electrostatic interaction of the polymer with bacterial cells, which is expressed as a systematic decrease in the logMIC value. The negative slope of the trend line indicates that an increase in the percentage of −NH_2_ groups is systematically associated with a downward trend in the logMIC value, pointing to an exploratory link between charge density and the bactericidal potency of soluble chitosan. Given that the strains studied are relatively diverse biologically, the fact that this clear linear trend persists exclusively in the soluble fraction is a particularly compelling finding, confirming that the presence of protonated amino groups remains the primary factor determining antimicrobial efficacy once diffusion barriers are eliminated.

However, the potential biological trade-off associated with the use of chitosans with an exceptionally high DD (>95%) must be taken into account. Although a high density of protonated amino groups significantly reduces the MIC against pathogens, it simultaneously enhances nonspecific electrostatic interactions between the polymer and mammalian cell membranes. This cationic charge density can lead to concentration-dependent cytotoxicity and alterations in cellular uptake, as previously demonstrated by Huang et al. (2004) and Park et al. (2011) [31,32]. Therefore, striking a balance between the antimicrobial benefits resulting from a high DD and biocompatibility remains a key challenge in the design of biomaterials for clinical applications.

Another physicochemical parameter that may influence the antibacterial activity of chitosan is the polydispersity index (PDI), which reflects the molecular weight distribution in the sample. A sample with a high degree of polydispersity may contain low-molecular-weight fractions that interact with microbial membranes differently than chains with higher molecular weights. Unfortunately, the vast majority of studies included in this review did not report PDI values or lacked methodological consistency in their determination. Consequently, we were unable to include PDI in our quantitative regression analysis, which represents a current limitation of the literature data and underscores the urgent need for more standardized characterization of polymers in future studies.

While DD plays the primary role, the data indicate that after simultaneously adjusting for MW and DD for the soluble fraction (DD > 60%), MW does not demonstrate a statistically significant independent effect on logMIC within the global model (*p* = 0.156). This suggests that variations in MW do not systematically alter chitosan antimicrobial effectiveness when treated as a single, global pool. However, the potential secondary or group-specific role of MW is further explored through the stratified analyses presented in the Supplementary Materials. The literature often assumes that lower molecular weight promotes higher antimicrobial activity, but the quantitative synthesis and statistical modelling showed the opposite trend [17,33].

Consequently, because MW lacks independent statistical significance, no directional or enhancing effect can be mathematically inferred from its global model coefficient, highlighting that the widespread literature assumption regarding the linear impact of MW is not supported by this overarching data aggregation. In contrast to these findings, the study by Hui et al. does not reveal a clear trend suggesting that composites of chitosan with low or high MW had different antimicrobial effects [34]. Other authors suggest that chitosans with low to medium MW (4–10 kDa) exhibit the strongest antibacterial activity, while oligomers are almost inactive, and chitosans with very high MW may exhibit reduced bioactivity, with few exceptions [15,20,35]. However, confirming the potential of larger molecules, it has been noted that chitosan exhibits a stronger antibacterial effect than its oligoderivatives [36]. The mechanism behind this phenomenon is not fully understood. However, two hypotheses dependent on the chain length may be considered:

- Low molecular weight chitosan (LMWC): Demonstrates an ability to penetrate the cell walls of microorganisms and the cell membrane due to its smaller hydrodynamic volume. Once inside the cytoplasm, LMWC interacts electrostatically with negatively charged intracellular macromolecules, such as DNA and RNA, thereby directly inhibiting transcription, protein synthesis, and key metabolic pathways [37,38].
- High molecular weight (HMWC): Abundant positively charged amino groups in HMWC interact electrostatically with the negatively charged cell wall. This leads to the formation of a dense polymer coating that disrupts normal cell metabolism, and its action focuses on binding essential metals, preventing the flow of nutrients, and modifying cell permeability [37,38].

An effort was also made to separately create regression models for Gram-positive (*n* = 46) and Gram-negative (*n* = 53) bacteria to evaluate the robustness and consistency of the structural predictors across these distinct biological subgroups. For both Gram-negative and Gram-positive bacteria, slight shifts in the adjusted R^2^ values to 0.38 and 0.37 were observed, respectively. While this variation could tentatively point to sample heterogeneity introduced by the inclusion of fungal datasets, the descriptive metrics summarized in Table 3 must be interpreted with extreme caution. The apparent differences in mean MW and logMIC between the two bacterial subtypes are minor, especially when evaluated against the remarkably large standard deviations and highly overlapping data ranges. Consequently, without formal comparative hypothesis testing, these parameters should be viewed strictly as overlapping exploratory distributions rather than distinct biological variations between the bacterial groups. To examine the behaviour of the regression parameters within each cohort, a forest plot was created showing unstandardized regression coefficients (B) along with their corresponding 95% confidence intervals (CI) (Figure 11). Statistical analysis showed that the DD is a highly significant negative predictor for logMIC values in both Gram-positive and Gram-negative bacteria. This consistent negative relationship suggested that the density of charge-carrying amine groups serves as a universal, dominant structural factor correlated with a lower minimum inhibitory concentration with respect to both types of cell walls. In sharp contrast to this, the 95% confidence intervals for the mean MW in both the Gram-positive and Gram-negative bacterial models closely surround or directly intersect the vertical reference line at zero, indicating negligible, statistically insignificant regression coefficients.

This graphical representation further supports the trend that, within the limits of the model’s moderate explanatory power and after accounting for the primary association with DD, variability in the average molecular weight does not make a statistically significant, independent contribution to this exploratory framework.

## 5. Conclusions

Insoluble polymer fractions (set as DD < 60%) can interfere with MIC determination and had to be excluded from the study to ensure free diffusion. The degree of deacetylation (DD) is the main factor influencing the biocidal activity of soluble chitosan (*p* < 0.0001), and the data analysis revealed that every 1% increase in DD statistically reduces the logMIC value by an average of 0.040 (95% CI: 0.029–0.050). MW did not emerge as a statistically significant independent predictor within our global model (*p* = 0.156), indicating that DD is the key driver of efficacy among soluble chitosans. The standardization of future chitosan-based biomaterials must focus on optimizing the degree of deacetylation to ensure both antimicrobial activity and biocompatibility. It must be noted that this analysis considered average MW without accounting for molecular weight distribution (polydispersity), which likely affects MIC results; however, most literature sources omit distribution data, making such an inclusion currently unfeasible. By filling the identified knowledge gaps regarding the precise physicochemical parameters influencing optimal efficacy, this study provides a concrete framework that facilitates the targeted design of advanced biomaterials. Ultimately, our findings enable researchers and engineers to consciously tailor chitosan-based formulations for specific applications requiring reliable biocidal, bactericidal, or antifungal properties.

## Figures and Tables

**Table 1:** Quantitative and percentage analysis of the tested strains at the species level.

No.	Genus Name	Species Epithet	Gram Staining	Number of Samples	Percentage Share
1	*Staphylococcus*	*aureus*	Positive	31	24.4%
2	*Escherichia*	*coli*	Negative	21	16.5%
3	*Pseudomonas*	*aeruginosa*	Negative	8	6.3%
4	*Streptococcus*	*mutans*	Positive	6	4.7%
5	*Bacillus*	*subtilis*	Positive	5	3.9%
6	*Candida*	*albicans*	N/A	5	3.9%
7	*Roseburia*	*intestinalis*	Negative	4	3.1%
8	*Bacteroides*	*thetaiotaomicron*	Negative	4	3.1%
9	*Streptococcus*	*sobrinus*	Positive	4	3.1%
10	*Bacteroides*	*vulgatus*	Negative	4	3.1%
11	*Clostridium*	*beijerinckii*	Positive	4	3.1%
12	*Faecalibacterium*	*prausnitzii*	Positive	4	3.1%
13	*Clostridium*	*paraputrificum*	Positive	4	3.1%
14	Others			23	18.1%
	TOTAL			127	100%

**Table 2:** Predicted logMIC values derived from the multiple regression model for the soluble chitosan fraction (DD ≥ 60%) across selected molecular weights (MW).

MW (kDa)\DD (%)	50	100	200	400
60	3.93	3.91	3.87	3.80
65	3.73	3.71	3.67	3.60
70	3.53	3.51	3.47	3.40
75	3.33	3.31	3.27	3.20
80	3.13	3.11	3.07	3.00
85	2.93	2.91	2.87	2.80
90	2.73	2.71	2.67	2.60
95	2.53	2.51	2.47	2.40
100	2.33	2.31	2.27	2.20

**Table 3:** Descriptive statistics of the dataset (*n* = 99, excluding 7 fungi).

Parameter	Gram-Negative	Gram-Positive
N	53	46
MWmean	147.4	165.78
SD	179.3	205.86
Range (MWmin-Mwmax, kDa)	6.00–624.00	6.00–700.00
DD mean (%)	83.54%	83.04%
DD mean SD	9.87%	10.40%
DD range (Min–Max, %)	66.00–100.00%	66.00–100.00%
LogMIC		
LogMICmean	2.93	3.09
LogMIC SD	0.66	0.62
LogMIC Range (Min–Max)	1.00–3.88	1.59–4.18

## Data Availability

The data presented in this study are available in the article and Supplementary Materials.

## Supplementary Materials

The following supporting information can be downloaded at: https://www.mdpi.com/article/10.3390/molecules31142412/s1, Table S1: The search strategy with respect to individual database; Table S2: Microorganisms considered based on Gram staining results and systematic classification; Table S3: Analysis of missing methodological data and experimental variables in the collected dataset from the literature on chitosan (*n* = 106); Figure S1: Series of diagnostic graphs of multiple regression for MW and DD for Gram-negative bacteria (*n* = 53); Figure S2: Series of diagnostic graphs of multiple regression for MW and DD for Gram-positive bacteria (*n* = 46).
