# Supplementary material for: Factors Determining the Antimicrobial Effectiveness of Chitosan: A Critical Analysis of the Impact of Molecular Weight and Degree of Deacetylation
Source: Molecules. 2026 Jul 9;31(14):2412. doi: 10.3390/molecules31142412 (PMC13413926; doi:10.3390/molecules31142412)
Supplement: Supplementary file 1 [file molecules-31-02412-s001.zip › molecules-4371258-supplementary.pdf]

Factors Determining the Antimicrobial Effectiveness of Chitosan: A Critical Analysis of the Impact of Molecular Weight and Degree of Deacetylation

Karolina Czajkowska [1], Maciej Rybicki [2], Karol Kamil Kłosiński [1,*] and Radosław Aleksander Wach [3,*]

[1] Department of Biomedicine and Experimental Surgery, Faculty of Medicine, Medical University of Lodz, Narutowicza 60, 90-136 Lodz, Poland; karolina.czajkowska@umed.lodz.pl
[2] Plastic, Reconstructive and Aesthetic Surgery Clinic, Institute of Surgery, Medical University of Lodz, Dr. Stefana Kopcinskiego 22, 90-153 Lodz, Poland; maciej.rybicki@stud.umed.lodz.pl
[3] Institute of Applied Radiation Chemistry, Faculty of Chemistry, Lodz University of Technology,
Wroblewskiego 15, 93-590 Lodz, Poland
* Correspondence: karol.klosinski@umed.lodz.pl (K.K.K.); radoslaw.wach@p.lodz.pl (R.A.W.)

Supplementary Table S1. The search strategy with respect to individual database

	String
PubMed/MEDLINE	*("Chitosan"[Mesh] OR "chitosan"[Title/Abstract] OR "chitosans"[Title/Abstract] OR "poly-D-glucosamine"[Title/Abstract] OR "polyglucosamine"[Title/Abstract] OR "deacetylated chitin"[Title/Abstract] OR "chitin deacetylation products"[Title/Abstract] OR "poliglusam"[Title/Abstract] OR "beta-1,4-poly-D-glucosamine"[Title/Abstract])*<br><br>*AND*<br><br>*("Anti-Infective Agents"[Mesh] OR "Anti-Bacterial Agents"[Mesh] OR "Antifungal Agents"[Mesh] OR "antimicrobial"[Title/Abstract] OR "antibacterial"[Title/Abstract] OR "antifungal"[Title/Abstract] OR "microbicidal"[Title/Abstract] OR "microbiostatic"[Title/Abstract] OR "bactericidal"[Title/Abstract] OR "fungicidal"[Title/Abstract] OR "anti-infective"[Title/Abstract] OR "antimycotic"[Title/Abstract])*<br><br>*AND*<br><br>*("Bacteria"[Mesh] OR "Fungi"[Mesh] OR "Gram-Negative Bacteria"[Mesh] OR "Gram-Positive Bacteria"[Mesh] OR "Staphylococcus aureus"[Mesh] OR "Escherichia coli"[Mesh] OR "Pseudomonas aeruginosa"[Mesh] OR "Klebsiella pneumoniae"[Mesh] OR "Enterococcus faecalis"[Mesh] OR "Streptococcus"[Mesh] OR "Salmonella"[Mesh] OR "Candida albicans"[Mesh] OR "Aspergillus"[Mesh] OR "Listeria monocytogenes"[Mesh] OR "bacteria"[Title/Abstract] OR "fungi"[Title/Abstract] OR "yeasts"[Title/Abstract] OR "molds"[Title/Abstract] OR "moulds"[Title/Abstract] OR "Gram-positive"[Title/Abstract] OR "Gram-negative"[Title/Abstract] OR "Staphylococcus aureus"[Title/Abstract] OR "MRSA"[Title/Abstract] OR "Escherichia coli"[Title/Abstract] OR "E. coli"[Title/Abstract] OR "Pseudomonas aeruginosa"[Title/Abstract] OR "Klebsiella pneumoniae"[Title/Abstract] OR "Enterococcus faecalis"[Title/Abstract] OR "Streptococcus"[Title/Abstract] OR "Salmonella"[Title/Abstract] OR "Candida albicans"[Title/Abstract] OR "Aspergillus"[Title/Abstract] OR "Listeria monocytogenes"[Title/Abstract])*<br><br>*AND*<br><br>*("In Vitro Techniques"[Mesh] OR "in vitro"[Title/Abstract] OR "minimum inhibitory concentration"[Title/Abstract] OR "MIC"[Title/Abstract] OR "zone of inhibition"[Title/Abstract])*

	NOT<br><br>*("Nanoparticles"[Mesh] OR "nanoparticle*"[Title/Abstract] OR "nano-particle*"[Title/Abstract] OR "nanocomposite*"[Title/Abstract] OR "nanosphere*"[Title/Abstract] OR "nanogel*"[Title/Abstract] OR "nanoemulsion*"[Title/Abstract] OR "Silver"[Mesh] OR "silver"[Title/Abstract] OR "AgNPs"[Title/Abstract] OR "Gold"[Mesh] OR "gold"[Title/Abstract] OR "AuNPs"[Title/Abstract] OR "antibiotic*"[Title] OR "synergy"[Title] OR "synergistic"[Title] OR "Food Packaging"[Mesh] OR "food"[Title] OR "agriculture"[Title] OR "plant"[Title] OR "water treatment"[Title] OR "Review"[Publication Type] OR "Systematic Review"[Publication Type] OR "Meta-Analysis"[Publication Type])*
**Embase(OVID)**	*(chitosan:ti,ab OR chitosans:ti,ab OR 'poly-d-glucosamine':ti,ab OR polyglucosamine:ti,ab OR 'deacetylated chitin':ti,ab OR 'chitin deacetylation products':ti,ab OR poliglusam:ti,ab OR 'beta-1,4-poly-d-glucosamine':ti,ab) AND (antimicrobial:ti,ab OR antibacterial:ti,ab OR antifungal:ti,ab OR microbicidal:ti,ab OR microbiostatic:ti,ab OR bactericidal:ti,ab OR fungicidal:ti,ab OR 'anti-infective':ti,ab OR antimycotic:ti,ab) AND (bacteria:ti,ab OR bacterium:ti,ab OR fungi:ti,ab OR fungus:ti,ab OR yeasts:ti,ab OR molds:ti,ab OR moulds:ti,ab OR 'gram-positive':ti,ab OR 'gram-negative':ti,ab OR 'staphylococcus aureus':ti,ab OR mrsa:ti,ab OR 'escherichia coli':ti,ab OR 'e. coli':ti,ab OR 'pseudomonas aeruginosa':ti,ab OR 'klebsiella pneumoniae':ti,ab OR 'enterococcus faecalis':ti,ab OR streptococcus:ti,ab OR salmonella:ti,ab OR 'candida albicans':ti,ab OR aspergillus:ti,ab OR 'listeria monocytogenes':ti,ab) AND ('in vitro':ti,ab OR 'minimum inhibitory concentration':ti,ab OR mic:ti,ab OR 'zone of inhibition':ti,ab) NOT (nanoparticle*:ti,ab OR 'nano-particle*':ti,ab OR nanocomposite*:ti,ab OR nanosphere*:ti,ab OR nanogel*:ti,ab OR nanoemulsion*:ti,ab OR silver:ti,ab OR agnps:ti,ab OR gold:ti,ab OR aunps:ti,ab OR antibiotic*:ti,ab OR synergy:ti,ab OR synergistic:ti,ab OR food:ti,ab OR agriculture:ti,ab OR plant:ti,ab OR 'water treatment':ti,ab)*
**Scopus**	*TITLE-ABS-KEY (*<br><br>*("chitosan" OR "chitosans" OR "poly-D-glucosamine" OR "polyglucosamine" OR "deacetylated chitin" OR "chitin deacetylation products" OR "poliglusam" OR "beta-1,4-poly-D-glucosamine")*<br><br>*AND*<br><br>*("Anti-Infective Agents" OR "Anti-Bacterial Agents" OR "Antifungal Agents" OR "antimicrobial" OR "antibacterial" OR "antifungal" OR "microbicidal" OR "microbiostatic" OR "bactericidal" OR "fungicidal" OR "anti-infective" OR "antimycotic")*<br><br>*AND*<br><br>*("Bacteria" OR "Fungi" OR "Gram-Negative Bacteria" OR "Gram-Positive Bacteria" OR "Staphylococcus aureus" OR "Escherichia coli" OR "Pseudomonas aeruginosa" OR "Klebsiella pneumoniae" OR "Enterococcus faecalis" OR "Streptococcus" OR "Salmonella" OR "Candida albicans" OR "Aspergillus" OR "Listeria monocytogenes" OR "bacteria" OR "fungi" OR "yeasts" OR "molds" OR "moulds" OR "Gram-positive" OR "Gram-negative" OR "MRSA" OR "E. coli")*<br><br>*AND*<br><br>*("In Vitro Techniques" OR "in vitro" OR "minimum inhibitory concentration" OR "MIC" OR "zone of inhibition")*<br><br>*)*<br><br>*AND NOT TITLE-ABS-KEY ("nanoparticle*" OR "nano-particle*" OR "nanocomposite*" OR "nanosphere*" OR "nanogel*" OR "nanoemulsion*" OR "Silver" OR "AgNPs" OR "Gold" OR "AuNPs" OR "Food Packaging")*<br><br>*AND NOT TITLE ("antibiotic*" OR "synergy" OR "synergistic" OR "food" OR "agriculture" OR "plant" OR "water treatment")*<br>*AND (LIMIT-TO (DOCTYPE , "ar"))*

Web of Science Core Collection

TS=("chitosan" OR "chitosans" OR "poly-D-glucosamine" OR "polyglucosamine" OR "deacetylated chitin" OR "chitin deacetylation products" OR "poliglusam" OR "beta-1,4-poly-D-glucosamine")

AND

TS=("Anti-Infective Agents" OR "Anti-Bacterial Agents" OR "Antifungal Agents" OR antimicrobial OR antibacterial OR antifungal OR microbicidal OR microbiostatic OR bactericidal OR fungicidal OR "anti-infective" OR antimycotic)

AND

TS=(Bacteria OR Fungi OR "Gram-Negative Bacteria" OR "Gram-Positive Bacteria" OR "Staphylococcus aureus" OR "Escherichia coli" OR "Pseudomonas aeruginosa" OR "Klebsiella pneumoniae" OR "Enterococcus faecalis" OR Streptococcus OR Salmonella OR "Candida albicans" OR Aspergillus OR "Listeria monocytogenes" OR bacteria OR fungi OR yeasts OR molds OR moulds OR "Gram-positive" OR "Gram-negative" OR MRSA OR "E. coli")

AND

TS=("In Vitro Techniques" OR "in vitro" OR "minimum inhibitory concentration" OR MIC OR "zone of inhibition")

AND NOT

TS=(Nanoparticles OR nanoparticle OR nano-particle* OR nanocomposite* OR nanosphere* OR nanogel* OR nanoemulsion* OR Silver OR AgNPs OR Gold OR AuNPs OR "Food Packaging")*

AND NOT

TI=(antibiotic OR synergy OR synergistic OR food OR agriculture OR plant OR "water treatment")*

AND
DT=Article

Supplementary Table S2. Microorganisms considered based on Gram staining results and systematic classification

Group	Number of Samples	Percentage Share
**Gram-Positive Bacteria**	63	49.6%
**Gram-Negative Bacteria**	57	44.9%
**Fungi**	7	5.5%
**Summary**	127	

Supplementary Figure S1. Series of diagnostic graphs of multiple regression for MW and DD for Gram-negative bacteria (N=53).

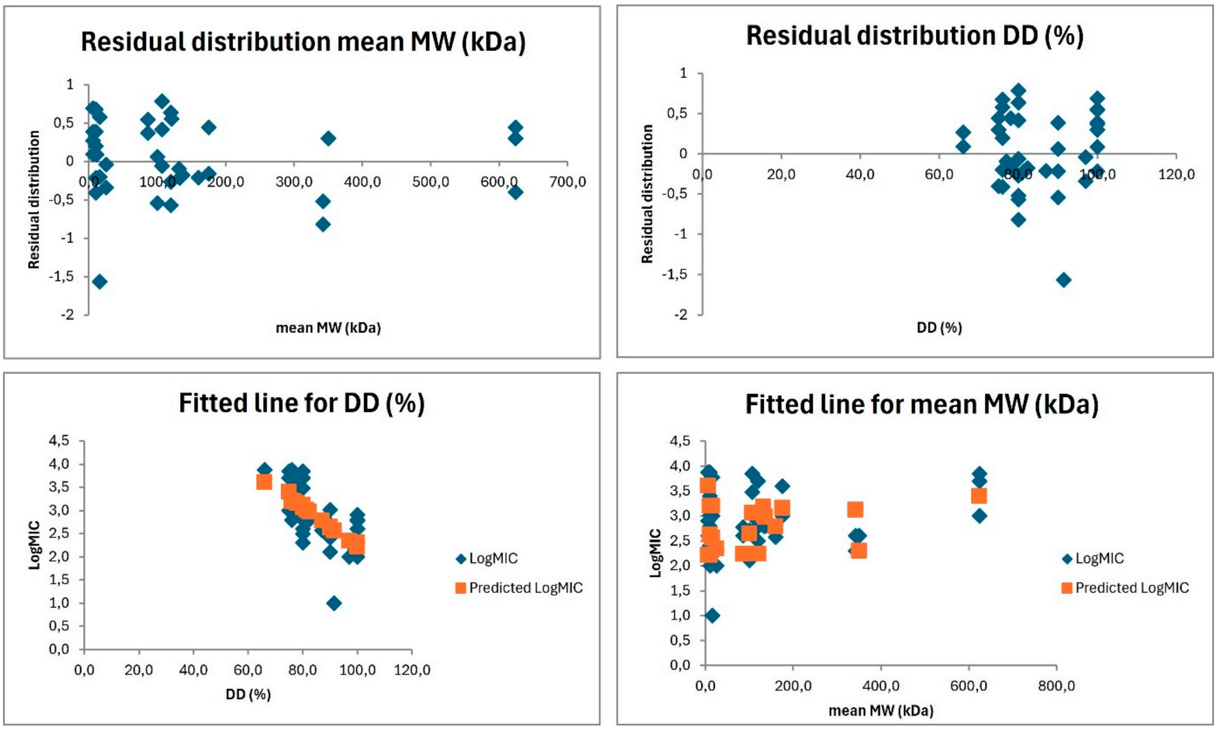

Supplementary Figure S2. Series of diagnostic graphs of multiple regression for MW and DD for Gram-positive bacteria (N=46).

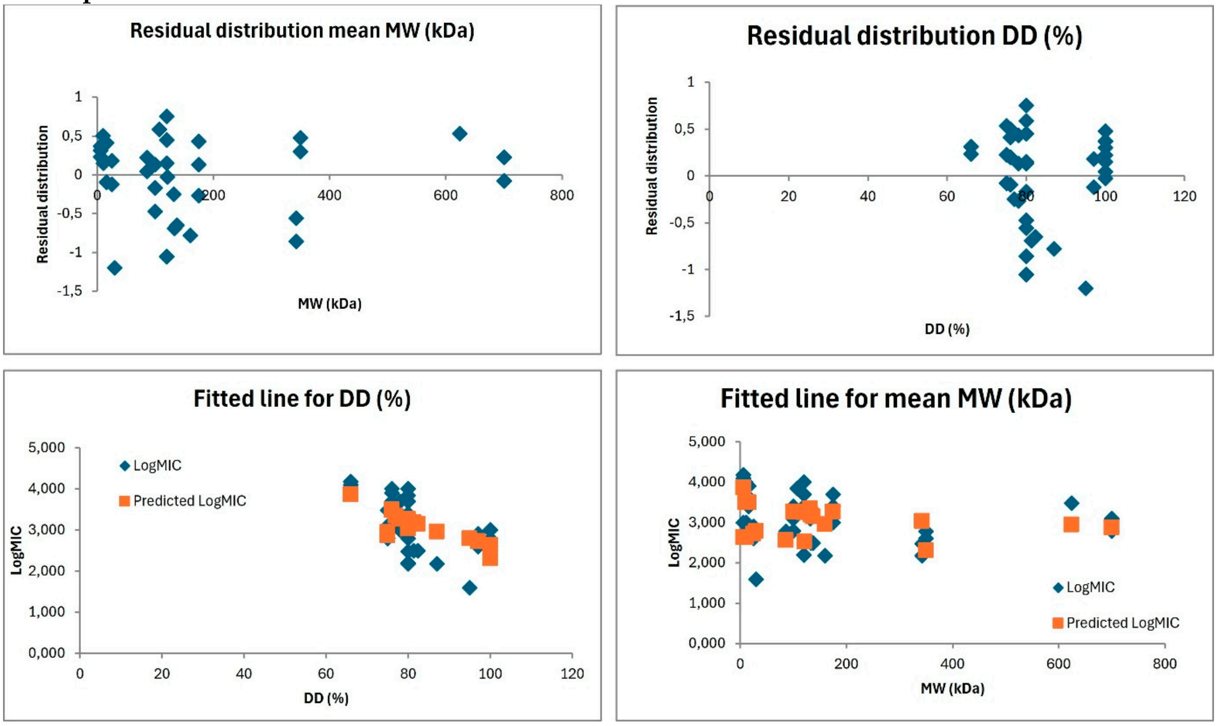

Supplementary Table S3. Analysis of missing methodological data and experimental variables in the collected dataset from the literature on chitosan (N = 106).

Variable	Reporting Status	Samples (n / 106)	% of dataset	Statistical Impact
**pH**	Not reported	80	75,5%	Excluded from the regression (significant gaps)
**Temperature**	Not reported	12	11,3%	Limited variation (mostly a constant 37°C)
**Incubation time**	Not reported	4	3,8%	High standardization (primarily 18h or 24h)
**Polydispersity**	No spread / Single value (MW mean)	70	66,0%	lack of data on the distribution
